# Identification of *avaC* from Human Gut Microbial Isolates that Converts 5AVA to 2-Piperidone

**DOI:** 10.1007/s12275-024-00141-0

**Published:** 2024-06-17

**Authors:** Qiudi Zhou, Lihui Feng

**Affiliations:** Institute of Pediatrics, Children’s Hospital of Fudan University, and Institutes of Biomedical Sciences, Fudan University, Shanghai, 200032 People’s Republic of China

**Keywords:** 2-piperidone, Gut microbiota metabolism, Cross-feeding, *avaC* (5-aminovaleric acid cyclase)

## Abstract

2-piperidone is a crucial industrial raw material of high-value nylon-5 and nylon-6,5. Currently, a major bottleneck in the biosynthesis of 2-piperidone is the identification of highly efficient 2-piperidone synthases. In this study, we aimed to identify specific strains among 51 human gut bacterial strains capable of producing 2-piperidone and to elucidate its synthetic mechanism. Our findings revealed that four gut bacterial strains, namely *Collinsella aerofaciens* LFYP39, *Collinsella intestinalis* LFYP54, *Clostridium bolteae* LFYP116, and *Clostridium hathewayi* LFYP18, could produce 2-piperidone from 5-aminovaleric acid (5AVA). Additionally, we observed that 2-piperidone could be synthesized from proline through cross-feeding between *Clostridium difficile* LFYP43 and one of the four 2-piperidone producing strains, respectively. To identify the enzyme responsible for catalyzing the conversion of 5AVA to 2-piperidone, we utilized a gain-of-function library and identified *avaC* (5-aminovaleric acid cyclase) in *C. intestinalis* LFYP54. Moreover, homologous genes of *avaC* were validated in the other three bacterial strains. Notably, *avaC* were found to be widely distributed among environmental bacteria. Overall, our research delineated the gut bacterial strains and genes involved in 2-piperidone production, holding promise for enhancing the efficiency of industrial biosynthesis of this compound.

**Supplementary Information:**

The online version contains supplementary material available at 10.1007/s12275-024-00141-0.

## Introduction

2-piperidone, a lactam molecule, derives its cyclization from ω-amino acids, serving as a fundamental scaffold for synthesizing various high-value chemical products, including nylon-5 and nylon-6,5 (Cheng et al., 2021; Frolov & Vereshchagin, 2023; Lubberink et al., 2023; Qiu et al., 2019; Zhang et al., 2022). Typically, 2-piperidone is prepared from 5-aminovaleric acid (5AVA) as the substrate in biosynthetic pathways (Zhao et al., 2023). Cyclization enzymes predominantly utilized for the production of 2-piperidone include ORF26, Act, and CaiC, sourced from bacteria (Chae et al., 2017; Zhang et al., 2017). ORF26, encoded by *Streptomyces aizunensis*, mediates the activation of 5AVA via ATP and facilitates the formation of 2-piperidone (Zhang et al., 2017). Act, belonging to the β-alanine CoA transferase family from *Clostridium propionicum*, catalyzes the activation of 5AVA using Acyl-CoA as a coenzyme, inducing its subsequent cyclization to 2-piperidone (Chae et al., 2017). CaiC, a crotonobetaine CoA ligase encoded by *Escherichia coli,* catalyzes the cyclization of 5-aminovaleric acid into 2-piperidone (Zhang et al., 2017). Notably, the cyclization step is rate-limiting in the biosynthesis of 2-piperidone (Zhao et al., 2023). The challenge lies in identifying novel enzymes capable of effectively producing 2-piperidone.

The human gastrointestinal (GI) tract hosts a dynamic and intricate community of gut bacteria, possessing millions of genes (Tierney et al., 2019; Zhang et al., 2010), engaged in various metabolic activities (Agus et al., 2018; Nicolas & Chang, 2019; Zhao et al., 2022). Previous studies have compared metabolites in the blood and tissues of germ-free and conventional mice, revealing that conventional mice exhibit a greater abundance of chemical species than germ-free mice (Zarei et al., 2022). However, the full potential of the human gut microbiota, which houses a vast array of genes encoding diverse small molecule synthetic proteins, remains largely untapped. Recognizing and investigating the metabolic enzyme resources carried by the gut microbiota is imperative. In a previous study, gnotobiotic mice were inoculated with human gut microbial consortia isolated from different developmental stages of a healthy infant (Feng et al., 2020). It was observed that the levels of 2-piperidone in the cecum and serum of mice inoculated with specific microbial consortia were significantly higher compared to germ-free mice (unpublished data from Lihui Feng), suggesting the potential production of 2-piperidone by human gut microbial isolates. Exploring the metabolic pathway within the gut microbiota responsible for producing 2-piperidone may unveil novel approaches for synthesizing 2-piperidone in indusial settings.

To identify the metabolic pathway of 2-piperidone, we analyzed the culture supernatant of 51 intestinal bacteria strains isolated from a human. From this analysis, we identified four strains capable of producing 2-piperidone from 5AVA: *Collinsella aerofaciens* LFYP39, *Collinsella intestinalis* LFYP54, *Clostridium bolteae* LFYP116, and *Clostridium hathewayi* LFYP18. Additionally, we established an in vitro cross-feeding system, where *Clostridium difficile* LFYP43 and the foregoing bacterial species produced 2-piperidone using proline as the substrate. Subsequently, we identified *avaC* as the gene responsible for converting 5AVA to 2-piperidone in the aforementioned strains through genomic DNA library screening and bioinformatic analysis. The determination of the metabolic pathway of 2-piperidone from the gut microbiota offers a promising approach to biosynthesizing 2-piperidone in industrial settings.

## Materials and Methods

### Reagents Used for This Research

All of the reagents used in this work are listed in Table S1.

### Bacterial Strains and Culture Condition

All of the strains used in this work are listed in Table S2. 51 gut bacterial strains previously derived from a healthy infant (Feng et al., 2020) were stocked at -80℃ in a 2 ml glass crimp top vial containing cell freezing medium (30% v/v glycerol, 70% v/v phosphate buffered saline, 0.5 g/L cysteine-HCl) until use. The 51 strains were cultured in LYHBHI broth (37 g/L BHI, 5 g/L yeast extract, 1 g/L cellobiose, 1 g/L maltose, 0.5 g/L L-cysteine-HCl, 5 mg/L hemin) or LYHBHI broth supplemented with 0.1% (w/v) soluble starch and 0.5% (w/v) partially purified porcine stomach mucin at 37℃ in an anaerobic chamber from Coy Laboratory Products. The optical density (OD_600_) was measured by GENESYS 30 spectrophotometer (Thermo Scientific). The gas composition used in the anaerobic chamber is 75% nitrogen, 20% carbon dioxide, and 5% hydrogen, and the hydrogen concentration in the chamber was maintained at ~ 2%.

All *E. coli* strains used in this work were cultured aerobically at 37 °C in LB (Luria–Bertani) broth or agar supplemented with kanamycin (100 mg/L), ampicillin (100 mg/L) or both of them as required overnight. Then the overnight culture was diluted 100-fold into the fresh LB with appropriate antibiotics.

### Genomic DNA Extraction

We isolated crude genomic DNA following standard protocols. Experimental procedures were conducted on ice. The cell pellet corresponding to 2 ml of culture with an OD_600_ of 1.0 was collected from the target strain in a tube by centrifugation at 17,000 × *g* for 2 min. Then, we added 200 μl of 0.5 mm glass beads, 500 μl of buffer A (0.2 M NaCl, 0.2 M Tris, and 0.2 M EDTA, adjusted to pH 8.0 with HCl), 210 μl of 20% (w/v) SDS and 500 μl of cold phenol–chloroform-isoamyl alcohol (25:24:1, pH 8.0) to each tube. The mixtures were homogenized using a Bionoon-192 High Throughput Tissue Grinder (BIONOON, Shanghai, China) at 60 Hz for 2 min. After centrifugation at 17,000 × *g* for 15 min at 4 °C, the supernatant from the uppermost layer was collected as the crude genomic DNA solution.

We then extracted pure genomic DNA from the crude genomic DNA solution. The crude genomic DNA solution was treated with RNase A (Takara) to remove RNA and purified using the QIAquick PCR Purification Kit (Qiagen). The purity and concentration of the pure DNA were analyzed using NanoDrop One (Thermo Scientific).

### Plasmid Construction and Cloning

Plasmids, synthetic DNA sequences, and primers used in this study are listed in Table S3. Plasmids were isolated using the Plasmid DNA Mini Kit I (Omega). Primers were designed using SnapGene v4.1.8 software (https://www.snapgene.com/) and ordered from Sangon Biotech. All DNA fragments used for plasmid construction were chemically synthesized by Genewiz. PCR reactions were performed using high-fidelity DNA polymerase (AB clonal). Plasmids were constructed by ligating the different fragments using the One Step Cloning Kit (Novoprotein) and chemically transformed into *E. coli* DH10B. The transformed *E. coli* DH10B cells were plated onto LB agar plates supplemented with appropriate antibiotics.

The biosensor plasmid (pLacSens) was constructed by ligating the PCR-linearized pEVS143 vector (primer no.1 and 2) and the synthetic DNA fragment 1 (Table S3). The pLacSens plasmid, harbored by *E. coli*, was selected on an LB agar plate supplemented with 100 mg/L of kanamycin. The constructed plasmid was verified by colony PCR and Sanger sequencing using primer no. 21–26.

Construction of production plasmids. The positive control plasmids and production plasmids carrying *avaC* homologous genes from bacterial species from various environments were constructed by ligating the synthetic DNA fragments (Table S3) respectively to the PCR-linearized pZE12 vector (primer no. 3 and 4). The negative control plasmid (pZE12 empty vector) was generated by ligating two PCR-linearized pZE12 plasmid vectors together (primer no. 5, 6 and primer no. 7, 8). Additional production plasmids were constructed by individually ligating the PCR-linearized targeted DNA sequences to the PCR-linearized pZE12 vector (primer no. 3 and 4). Targeted DNA sequences were cloned from the genomic DNA of strains, including “*CILFYP54_00697*” (primer no.9 and 10), “2931 bp” (primer no. 17 and 18), “*ypdA*” (primer no. 19 and 20) and “*CILFYP54_00697* + *ypdA*” (primer no. 9 and 18) from *C. intestinalis* LFYP54; “*CALFYP39_00071*” (primer no. 11 and 12) from *C. aerofaciens* LFYP39; “*CHLFYP18_04700*” (primer no. 13 and 14) from *C. hathewayi* LFYP18; “*CBLFYP116_02895*” (primer no. 15 and 16) from *C. bolteae* LFYP116. The production plasmids, carried by *E. coli*, were screened out on an LB agar plate supplemented with 100 mg/L of ampicillin. The constructed plasmids were verified by colony PCR and Sanger sequencing using primer no. 27 and 28.

### Production Plasmid Library Construction

We employed a gain-of-function strategy to construct a production plasmid library, following a previously described method (Zimmermann et al., 2019). Briefly, pure genomic DNA was extracted from *C. intestinalis* LFYP54 grown to early stationary phase in LYHBHI. 10 μg of the pure genomic DNA were uniformly apportioned into ten 0.6 mL EP tubes, each containing a concentration of 10 ng/μl. Subsequently, the contents were pelleted and incubated on ice for 10 min. Each sample was sheared by sonication (Diagenode) using a single duty cycle of 10 s on/90 s off at low amplitude. Following the collection of samples and gel electrophoresis, DNA fragments ranging from 2–8 kb were extracted from a 0.7% agarose gel using the QIAquick Gel Extraction Kit (Qiagen). The gel-recovered fragments were cloned into the linearized pZE12 vector (primer no. 3 and 4) by blunt-end ligation enzyme (Epicentre FastLinkTM kit) and ligation products in the range of 4–10 kb were extracted from a 0.7% agarose gel using the QIAquick Gel Extraction Kit (Qiagen).

### Targeted Gene Screening Assay

The recovered ligation products were transformed into *E. coli* with pLacSens electrocompetent cells. From the resulting single clones grown on LB agar plates, we selected and transferred them to a 384-well plate containing LB medium supplemented with 100 mg/L kanamycin and 100 mg/L ampicillin. Overnight cultures from the 384-well plate were then diluted at a 1:100 (v/v) ratio into 96-well plates containing LB medium, with or without 5AVA. Furthermore, the LB medium was supplemented with 500 μM isopropyl β-D-1-thiogalactopyranoside (IPTG), 100 mg/L kanamycin, and 100 mg/L ampicillin. Subsequently, the plates were incubated for 24 h. GFP and OD_600_ values were measured using the Synergy LX Multimode Reader (BioTek). The fold change of [GFP (AU)/OD_600_] was determined as $\frac{[\frac{{\text{GFP}}}{{\text{OD}}600}]\mathrm{\;of\;the\;culture\;with\;}5{\text{AVA}}}{[\frac{{\text{GFP}}}{{\text{OD}}600}]\mathrm{\;of\;the\;culture\;without\;}5{\text{AVA}}}$ for the original clonal subculture in LB medium. Strains with the highest values of fold change of [GFP (AU)/OD_600_] were selected for further LC–MS/MS analysis.

### Precursor Metabolite Incubation with Resting Cell Suspensions

Precursor metabolite incubation experiments were performed following a previously described protocol (Dodd et al., 2017). All procedures were carried out in the anaerobic chamber. Briefly, overnight cultures of each gut bacterial strain were diluted 1000-fold into the fresh LYHBHI broth and grown to the early stationary phase. Cell pellets equivalent to 20 ml of OD_600_ = 1.0 from each strain for co-culture strains were harvested by centrifugation at 4,347 × *g* for 10 min. The cell pellets were washed twice with PBS, followed by resuspension in 4 ml of PBS. The cell resuspension was incubated at 37 °C for 30 min to allow the cells to consume a majority of the remaining nutrients in LYHBHI. Subsequently, the resuspension was divided equally into two portions and transferred to versatility tubes, with one portion receiving 1 mM (final concentration) metabolic precursor and the other untreated as negative control. Then we continued incubating resuspensions for 1 h at 37 °C. Finally, the supernatant of the bacterial incubation was collected by centrifugation at 17,000 × *g* for 10 min and analyzed for metabolites using LC–MS/MS. For *C. bolteae* LFYP116, the strain supernatant was separated from the strain pellet by filtering using a 0.22 μm filter (Millipore).

### Liquid Chromatography-Tandem Mass Spectrometry (LC–MS/MS) Quantitative Analysis

All samples were centrifuged at 17,000 × *g* for 20 min before detection by LC–MS/MS. A Shimadzu LC (Shimadzu) with a Nucleodur C18 ISIS column (2 × 250 mm; Welch) coupled to an AB SCIEX 4000 linear ion trap mass spectrometer (SCIEX) was used to separate and detect the target compounds in samples. Two microliters of each sample were injected into the mobile phase via autosampler and separated at a flow rate of 0.2 ml/min. The mobile phase consisted of water with 0.1% (v/v) formic acid as eluent A and acetonitrile as eluent B. The gradient elution started with 100% eluent A for 4 min, followed by a linear gradient from 0 to 90% (v/v) eluent B in 7 min, and equilibrated with 90% eluent B for 2 min. Mass spectrometry was performed in positive ion mode under scan mode from 100 m/z to 400 m/z (For detailed parameters see Table S4). MRM parameters and retention times of the target compounds are shown in Table S5. The target compounds were quantified from calibration curves plotted with external standards. Some samples were analyzed by AB SCIEX 6500 triple-quadrupole liquid chromatography-mass spectrometry system (SCIEX).

### Statistical Analysis

We performed statistical analyses using GraphPad Prism 8.0 software (https://www.graphpad.com/). The mean ± standard deviation was presented for values in the results. The statistical significance of the analysis between the two groups was determined by the Student’s t-test (unpaired, two-tailed). For comparisons involving multiple groups, One-way ANOVA and Tukey’s multiple comparison test were used. The significance levels were represented as follows: ns, not significant; *p < 0.05; **p < 0.01; ***p < 0.001; ****p < 0.0001.

### 16S rDNA Phylogenetic Tree Construction

16S rRNA gene sequences (1249–1310 bp) were obtained in the previous study (Feng et al., 2020). The 16S rRNA gene sequences of 51 gut bacteria were aligned using the SILVA Aligner service (https://www.arb-silva.de/aligner) (Pruesse et al., 2012). The aligned sequences were imported into MEGA X v10.2.6 software (https://www.megasoftware.net/) (Kumar et al., 2018) to construct phylogenetic trees.

Evolutionary relationships between 51 bacterial strains were analyzed using Maximum Likelihood method. The evolutionary history was inferred by using the Maximum Likelihood method and Tamura-Nei model (Tamura & Nei, 1993). The tree with the highest log likelihood (-23,769.34) is shown. The percentage of trees in which the associated taxa clustered together is shown next to the branches. Initial tree(s) for the heuristic search were obtained automatically by applying Neighbor-Join and BioNJ algorithms to a matrix of pairwise distances estimated using the Tamura-Nei model and then selecting the topology with superior log likelihood value. This analysis involved 51 nucleotide sequences. There were a total of 1434 positions in the final dataset. Evolutionary analyses were conducted in MEGA X (Kumar et al., 2018).

### Homologous Gene Alignment

The information on genome sequencing, assembly, and annotation was provided in the previous study (Feng et al., 2020). The Nucleotide Basic Local Alignment Search Tool (BLASTn) (https://blast.ncbi.nlm.nih.gov/Blast.cgi) was used to search the homologous genes of *CILFYP54_00697* or assess the homology of the gene cluster (*CILFYP54_00696* to *CILFYP54_00698*) in the genomes of *C. bolteae* LFYP116, *C. aerofaciens* LFYP39 and *C. hathewayi* LFYP18.

The Protein Basic Local Alignment Search Tool (BLASTp) was used to search the NCBI GenBank database with the amino acid sequences of AvaC from *C. intestinalis* LFYP54, *C. aerofaciens* LFYP39, *C. hathewayi* LFYP18 and *C. bolteae* LFYP116 as queries. The protein sequences of identity > 50% and coverage > 80% were filtered in the search results, and the protein information was listed in Table S6.

## Results

### Identifying Gut Microbial Isolates that Produce 2-Piperidone from 5AVA

**Fig. 1:**
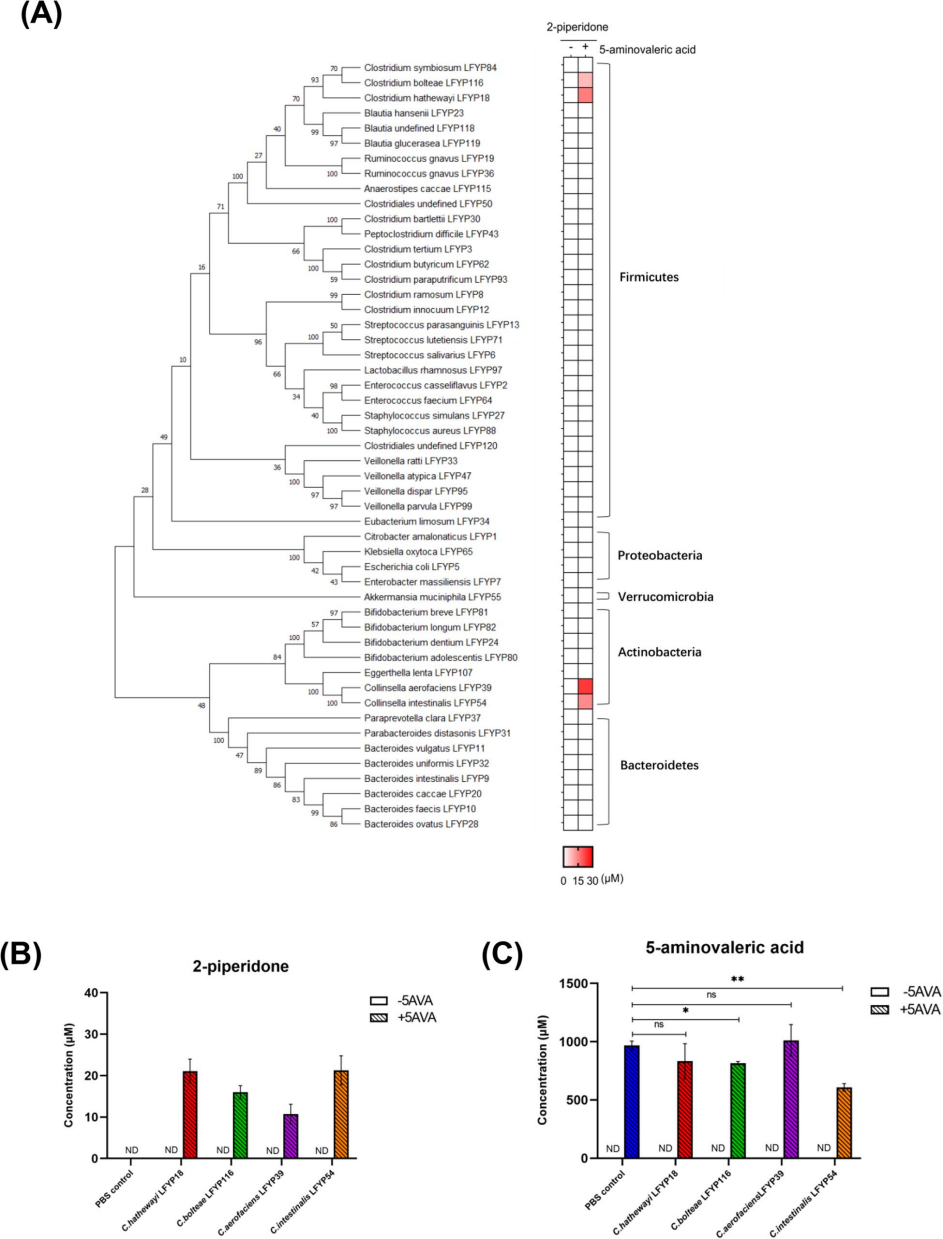
Four gut microbial species convert 5-aminovaleric acid (5AVA) to 2-piperidone. (**A**) Heat map showing the mean concentration of 2-piperidone in the supernatant from 51 gut bacterial strains incubated without or with 5AVA (n = 3 biological replicates). The phylogenetic tree of the 51 gut bacterial strains was constructed by Maximum Likelihood method and Tamura-Nei model based on 16S rDNA sequences. The percentage of trees in which the associated taxa clustered together is shown next to the branches. (**B**) Concentration of 2-piperidone in the supernatant of PBS control and the four gut microbial strains incubated without or with 1 mM 5AVA. Statistically significant differences between 2-piperidone concentrations in the four gut microbiota strains co-incubated with 5AVA were determined by One-way ANOVA and Tukey’s multiple comparison test. No significant differences were detected. (C) Concentration of 5AVA in the supernatant of PBS control and the four gut microbial strains incubated without or with 1 mM 5AVA. Statistically significant differences between 5AVA concentrations in the PBS with 5AVA control group and each of the four bacterial strains co-incubated with 5AVA were determined by Student’s t-test (unpaired, two-tailed). ns, not significant; *p < 0.05; **p < 0.01. For (B) and (C), error bars represent the standard error of mean from three biological replicates. ND, not detected

Based on the molecular structure of 2-piperidone and previous studies, we hypothesized that 5AVA could serve as a precursor of 2-piperidone. To ascertain which gut bacterial strains possess the ability to metabolize 5AVA to 2-piperidone whthin the human gut bacterial culture collection in the lab, we cultured 51 gut bacterial strains representing five major gut microbial phyla. Subsequently, we incubated each strain with or without 5AVA in vitro and quantified the concentration of 2-piperidone in the supernatant using LC–MS/MS (see ‘Materials and Methods’). Our investigation revealed that four bacterial species, namely *Collinsella intestinalis* LFYP54, *Clostridium bolteae* LFYP116, *Collinsella aerofaciens* LFYP39, and *Clostridium hathewayi* LFYP18, demonstrated the capacity to produce 2-piperidone when exposed to 5AVA (Fig. 1A). Phylogenetic analysis of the 51 intestinal bacterial strains based on the sequence of 16S ribosomal RNA genes revealed that the two strains of Collinsella, *C. intestinalis* LFYP54, and *C. aerofaciens* LFYP39, were clustered together at the same branch of the phylogenetic tree and were closely related, as were the two strains of Clostridium, *C. bolteae* LFYP116 and *C. hathewayi* LFYP18. However, the relationship between Collinsella and Clostridium is not adjacent (Fig. 1A).

To validate the aforementioned positive outcomes, we conducted additional independent experiments with three biological replicates involving *C. intestinalis* LFYP54, *C. bolteae* LFYP116, *C. aerofaciens* LFYP39, and *C. hathewayi* LFYP18. The results indicated that the concentration of 2-piperidone generated were 21.30 ± 6.02 μM, 15.99 ± 2.79 μM, 10.71 ± 4.10 μM, and 21.09 ± 5.00 μM, respectively, after co-incubation with 1 mM 5AVA (Fig. 1B). Notably, statistical analysis by One-way ANOVA and Tukey’s multiple comparison test revealed no significant difference between 2-piperidone levels produced by the four strains. The conversion rates of 5AVA to 2-piperidone were comparable across the four strains, at 2.2%, 1.6%, 1.1%, and 2.2%, respectively. Only *C. intestinalis* LFYP54 and *C. bolteae* LFYP116 exhibited significant consumption of 5AVA (Fig. 1C), suggesting its potential involvement in other metabolic activities.

### *Clostridium difficile* and the 2-Piperidone Producing Strain Together Produce 2-Piperidone from Proline by Metabolic Cross-Feeding in vitro

**Fig. 2:**
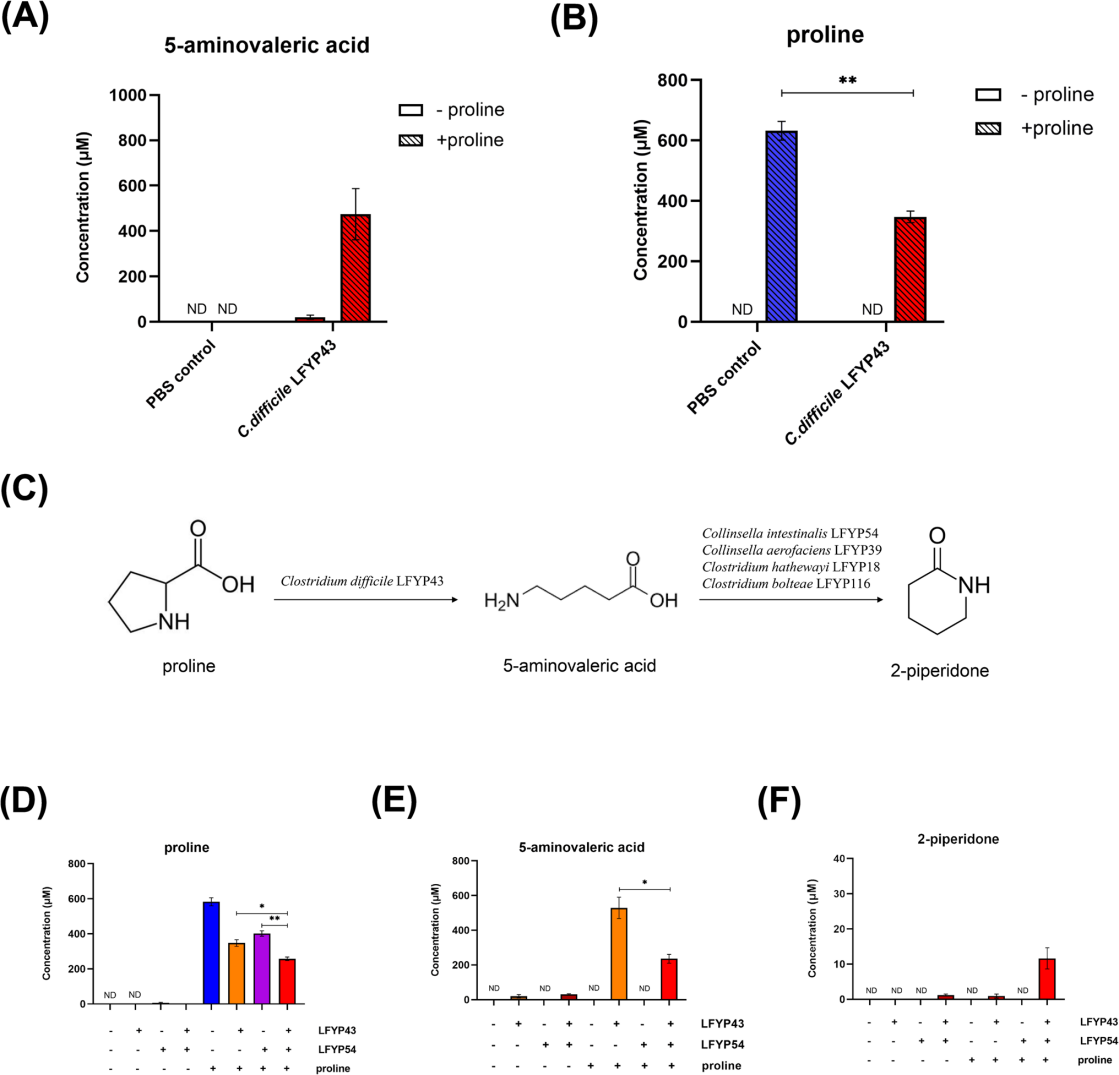
Gut microbial strains produce 2-piperidone from proline through cross-feeding in vitro. Concentration of 5AVA (**A**) and proline (**B**) in the supernatant of PBS control and *C. difficile* LFYP43 incubated without or with 1 mM proline. (**C**) A proposed cross-feeding pathway between *C. difficile* LFYP43 and the four 2-piperidone-producing strains. Concentration of proline (**D**), 5AVA (**E**), and 2-piperidone (**F**) in the supernatant of *C. difficile* LFYP43 and *C. intestinalis* LFYP54 under different incubation conditions. In (B), statistical analysis was conducted to compare the proline concentrations between the PBS with proline control and *C. difficile* LFYP43 incubated with proline. In (D) and (E), statistical analysis was conducted to compare the concentrations of proline (D) and 5AVA (E) under specific conditions. For (B), (D), and (E), the statistical significance was determined by using Student’s t-test (unpaired, two-tailed). *p < 0.05; **p < 0.01. For (A), (B), and (D–F), error bars represent the standard error of mean from three biological replicates. ND, not detected

The results above demonstrated that four human gut microbes possessed the capability to synthesize 2-piperidone from 5AVA. We aimed to investigate the origin of 5AVA, considering previous findings indicating its production by *Clostridium difficile* and *Clostridium sporogenes* from proline (Bouillaut et al., 2013; Liu et al., 2022). We postulated that *C. difficile* LFYP43 from our collection of gut bacterial strains could similarly convert proline into 5AVA. Employing the experimental procedures outlined earlier, we subjected *C. difficile* LFYP43 to incubation with proline. The outcomes revealed that *C. difficile* LFYP43 yielded 474.50 ± 195.60 μM of 5AVA upon incubation with 1000 μM proline (Fig. 2A). In control experiments where *C. difficile* LFYP43 was incubated without proline, minimal concentration of 5AVA were observed, measuring at 20.19 ± 16.82 μM (Fig. 2A). Concurrently, the level of proline decreased by approximately half during incubation with *C. difficile* LFYP43 (Fig. 2B).

Based on these findings, we hypothesized the occurrence of metabolic cross-feeding between *C. difficile* LFYP43 and one of the aforementioned bacterial species capable of producing 2-piperidone from 5AVA. Specifically, in co-incubation settings, proline could undergo metabolic conversion to 5AVA by *C. difficile* LFYP43, followed by the subsequent transformation of 5AVA into 2-piperidone by *C. intestinalis* LFYP54, *C. bolteae* LFYP116, *C. aerofaciens* LFYP39, or *C. hathewayi* LFYP18 (Fig. 2C). To assess this hypothesis, we conducted analogous incubation experiments with both *C. difficile* LFYP43 and *C. intestinalis* LFYP54 first, with proline introduced as the substrate in vitro. LC–MS/MS analysis of the supernatant revealed a significant increase in proline consumption when both *C. intestinalis* LFYP54 and *C. difficile* LFYP43 were co-incubated with 1000 μM proline, compared to incubation with *C. difficile* LFYP43 alone (Fig. 2D). Meanwhile, co-incubation of *C. difficile* LFYP43 and *C. intestinalis* LFYP54 with 1000 μM proline resulted in a notably reduced production of 5AVA compared to incubation with *C. difficile* LFYP43 alone, likely attributable to the further metabolism of 5AVA by *C. intestinalis* LFYP54 (Fig. 2E). Notably, incubation of proline with either *C. intestinalis* LFYP54 or *C. difficile* LFYP43 alone scarcely yielded 2-piperidone. However, co-incubation of *C. difficile* LFYP43 and *C. intestinalis LFYP54* with 1000 μM proline led to the production of 11.62 ± 5.25 μM of 2-piperidone (Fig. 2F). Analogous co-incubation experiments were performed between *C. difficile* LFYP43 and *C. aerofaciens* LFYP39, *C. hathewayi* LFYP18, or *C. bolteae* LFYP116. 2-piperidone were produced at 2.63 ± 0.07 μM, 5.88 ± 0.38 μM, 0.79 ± 0.11 μM respectively as analyzed by LC–MS/MS (Fig. S1). These outcomes provided evidence supporting the existence of cross-feeding interactions between *C. difficile* LFYP43 and one of the four 2-piperidone producing strains, wherein these strains collaboratively produced 2-piperidone utilizing proline as the substrate.

### *avaC* from *C. intestinalis* LFYP54 Converts 5AVA to 2-Piperidone

The above study revealed four bacterial strains that could convert 5AVA to 2-piperidone. Following this finding, our aim was to identify the gene responsible for catalyzing this conversion. Previous reports indicated that ORF26 in *Streptomyces aizunensis*, β-alanine CoA transferase (Act) in *Clostridium propionicum,* and carnitine CoA ligase (CaiC) in *Escherichia coli* were involved in cyclizing 5AVA to form 2-piperidone (Chae et al., 2017; Zhang et al., 2017). We conducted sequence alignments of *orf26*, *act*, and *caiC* with the whole-genome sequencing data of the four bacterial strains. However, no homologous sequence was found, suggesting that these newly identified bacterial strains utilize a distinct mechanism.

**Fig. 3:**
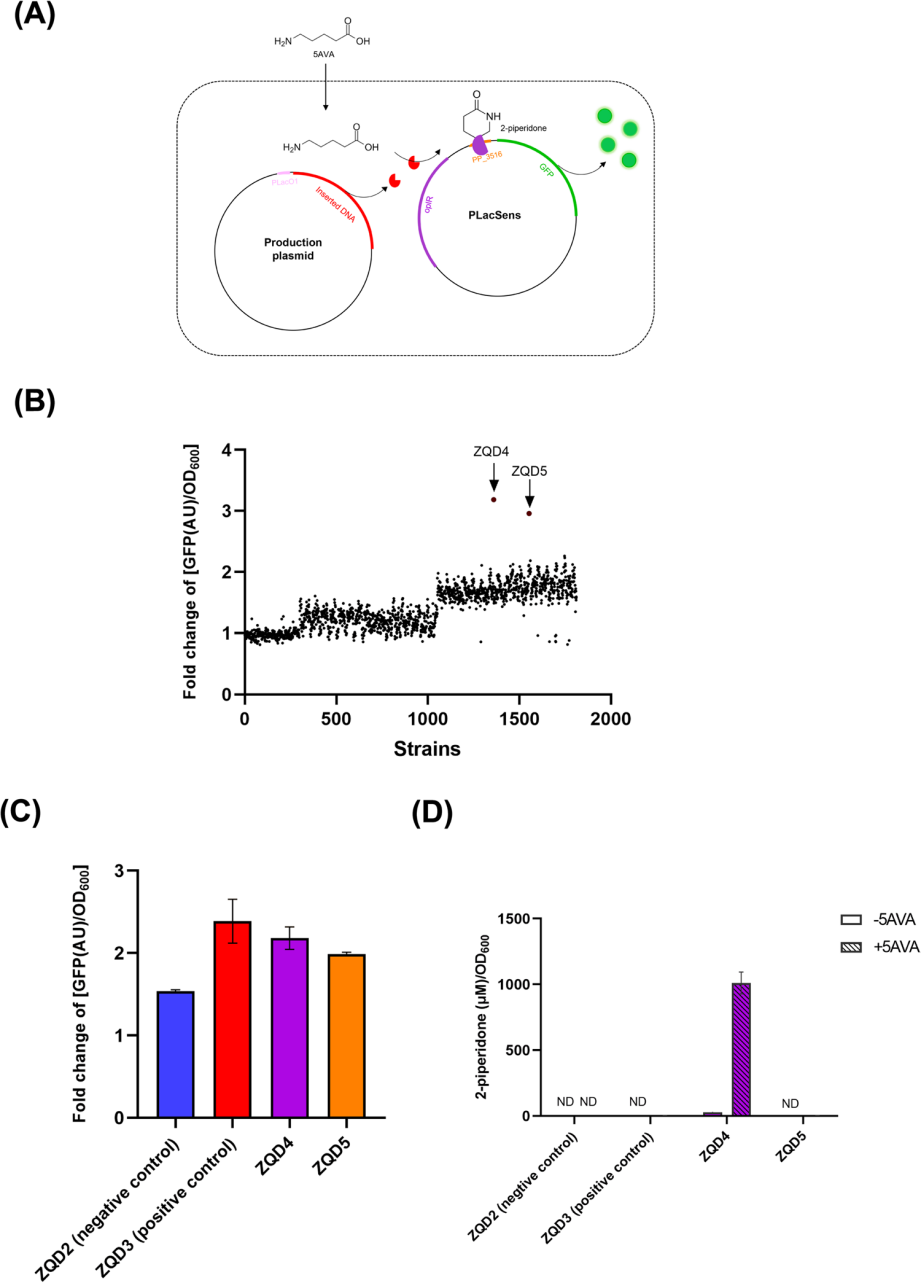
Identification of the genomic sequence in *C. intestinalis* LFYP54 which converts 5AVA to 2-piperidone. (**A**) Schematic plot illustrating the principle of the two-plasmid biosensor screening system. (**B**) Fold change of [GFP(AU)/OD_600_] values (see Methods for definition) from *E. coli* single colonies. Colonies with obvious higher fold-change values than the others were indicated by the arrows (ZQD4 and ZQD5). (**C**) Fold change of [GFP(AU)/OD_600_] values from the negative and positive control strains, ZQD4 and ZQD5. (**D**) Concentration of 2-piperidone per OD_600_ cell in the supernatant when strains were cultured without or with 5 mM 5AVA. For (C) and (D), error bars represent the standard error of mean from three biological replicates. ND, not detected

To identify the gene product responsible for producing 2-piperidone from 5AVA in the four bacterial strains, we employed a previously described two-plasmid biosensor system (Thompson et al., 2020). The system, utilized in *E. coli,* consisted of a biosensor plasmid (pLacSens) and a production plasmid. The pLacSens expressed green fluorescent protein (GFP) upon detecting 2-piperidone through the transcription factor OplR. The production plasmid was inserted with random DNA sequences under the control of the PLlacO1 promoter. If the DNA sequence inserted in the production plasmid was capable of producing 2-piperidone from 5AVA, green fluorescence would be observed (Fig. 3A). To assess the sensitivity of the biosensor plasmid for detecting 2-piperidone, various concentrations of 2-piperidone were added to the culture medium of *E. coli* harboring the pLacSens plasmid, and GFP expression was measured. The results indicated a 2.15 ± 0.05 fold increase in GFP fluorescence when 1 μM of 2-piperidone was added compared to the control group without adding 2-piperidone. Furthermore, GFP fluorescence reached saturation when 100 μM of 2-piperidone was added, which corresponds to a 6.54 ± 0.09 fold increase compared to control group (Fig. S2, squares). Subsequently, two control strains were constructed. The negative control strain carried pLacSens and an empty production plasmid, whereas the positive control carried pLacSens and a production plasmid inserted with *orf26*. 2-piperidone induction of GFP production was observed in *E. coli* carrying the two-plasmid system (Fig. S2). To assess the system's efficacy, the negative and positive control strains were cultured in LB medium with and without 5AVA, and GFP and 2-piperidone levels were measured (Fig. S3). In the positive control strain, the GFP signal increased 1.35 ± 0.06 fold with 5AVA compared to without 5AVA, while in the negative control strain, the GFP signal increased 0.78 ± 0.04 fold (Fig. S3A). LC–MS/MS analysis revealed that the positive control strain produced 14.11 ± 2.78 μM 2-piperidone after incubation with 5000 μM 5AVA, whereas the negative control strain did not produce detectable levels of 2-piperidone (Fig. S3B). These results established the validity of the two-plasmid screening system.

The efficiency of transforming 5AVA to 2-piperidone was similar among the four bacterial strains (Fig. 1B). Hence, one of these strains, *C. intestinalis* LFYP54, was randomly chosen for screening of target genes. Genomic DNA fragments from *C. intestinalis* LFYP54 were inserted into an empty production plasmid (see the section ‘Materials and Methods’). From the resulting 1811 colonies grown on antibiotic selective media, those colonies were cultured both with and without 5AVA, and the GFP signal was assessed for each culture. The fold change of [GFP (AU)/OD_600_] was calculated for each colony (see the section ‘Materials and Methods’). Among these colonies, strains ZQD4 and ZQD5 exhibited higher fold change of [GFP (AU)/OD_600_] values compared to the other colonies (Fig. 3B). Subsequently, ZQD4 and ZQD5 were isolated and replication experiments confirmed the initial screening results (Fig. 3C). Additionally, LC–MS/MS analysis confirmed the production of 2-piperidone from ZQD4 and ZQD5. Surprisingly, only ZQD4 produced a high concentration of 2-piperidone after co-incubation with 5AVA, while ZQD5 only produces a minimal concentration of 2-piperidone (Fig. 3D). Hence, our focus shifted to ZQD4. It was noteworthy that the average concentration of 2-piperidone produced by ZQD4 after incubating with 5 mM of 5AVA was approximately 50-fold greater than that produced by the positive control strain within the same batch of experiments. This suggests that the gene carried by ZQD4 had a much higher ability to convert 5AVA to 2-piperidone compared to the previously reported *orf26*.

**Fig. 4:**
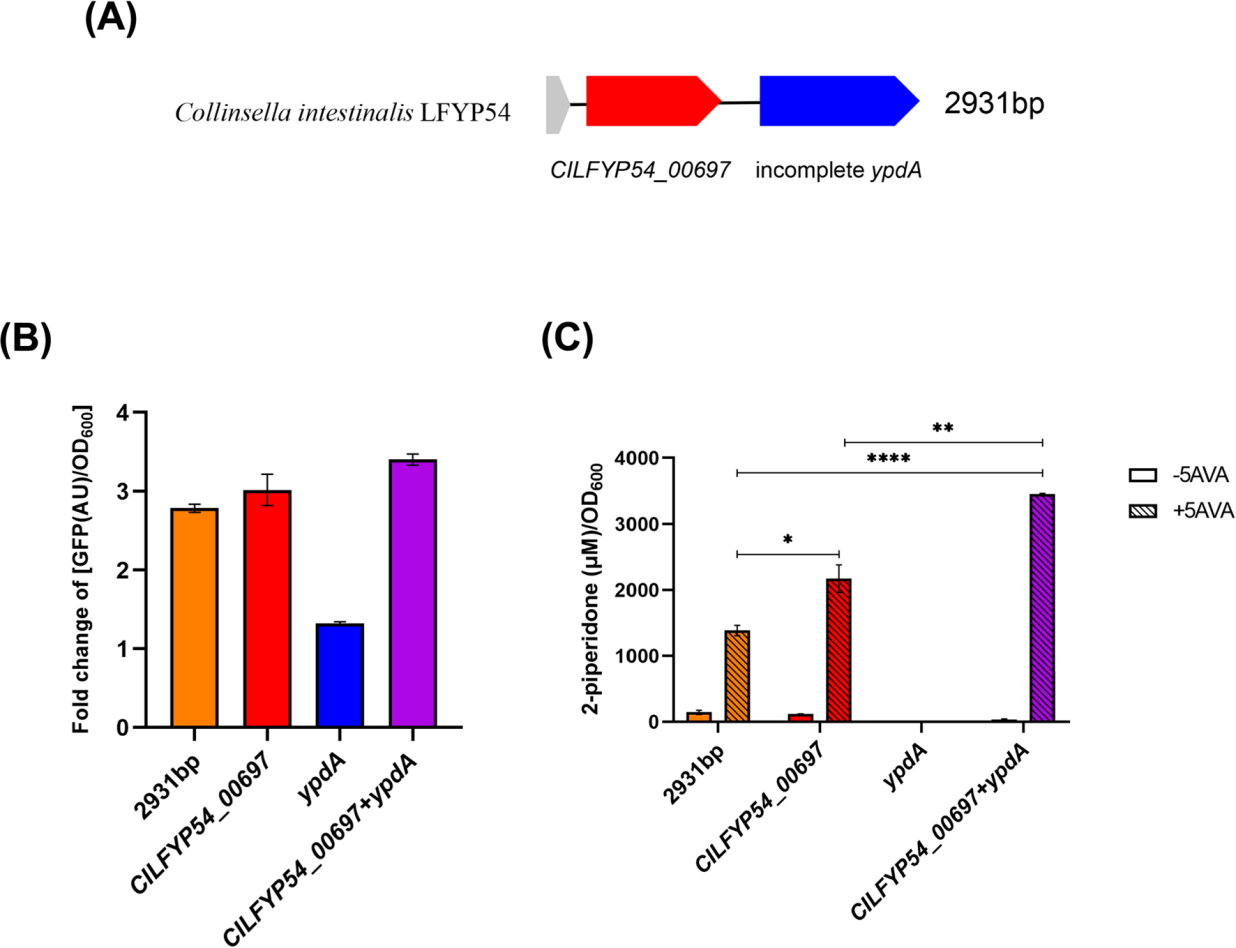
*avaC* in *C. intestinalis* LFYP54 converts 5AVA to 2-piperidone. (**A**) Schematic plot showing the 2931 bp DNA sequence inserted into the production plasmid of strain ZQD4. *CILFYP54_00697* encodes a hypothetical protein and *ypdA* encodes the sensor histidine kinase YpdA. (**B**) Fold change of [GFP(AU)/OD_600_] of *E. coli* strains with the two-plasmid biosensor system heterologously expressing the different DNA sequences as indicated. (**C**) Concentration of 2-piperidone per OD_600_ cell in the supernatant of strains cultured without or with 5 mM 5AVA. Statistically significant differences between 2-piperidone concentrations in the strains carrying “*2931 bp*”, “*CILFYP54_00697*” and “*CILFYP54_00697* + *ypdA*”, respectively, and co-incubated with 5AVA, were determined by One-way ANOVA and Tukey’s multiple comparison test. *p < 0.05; **p < 0.01; ****p < 0.0001. For (B) and (C), error bars represent the standard error of mean from three biological replicates

The DNA sequence inserted into the production plasmid of ZQD4 was sequenced and aligned with the whole-genome sequence of *C. intestinalis* LFYP54. The results indicated that the 2931 bp inserted DNA fragment primarily contained two genes, *CILFYP54_00697* and an incomplete *ypdA* (Fig. 4A). *CILFYP54_00697* encodes a hypothetical protein annotated as an amidohydrolase family protein, while *ypdA* encodes a sensor histidine kinase (YpdA). To determine which gene could catalyze the conversion of 5AVA to 2-piperidone, each gene was individually cloned into the production plasmid. When expressing *CILFYP54_00697* heterologously, either independently or in combination with *ypdA*, we observed an approximately threefold increase in [GFP(AU)/OD_600_] in the presence of 5AVA compared to its absence, similar to the expression of the 2931 bp sequence. However, expressing *ypdA* alone resulted in only a modest increase in GFP (Fig. 4B). LC–MS/MS analysis revealed that expression of *CILFYP54_00697* produced 2.17 ± 0.36 mM 2-piperidone in the supernatant when 5 mM 5AVA was added to the medium, whereas expressing *ypdA* alone produced a negligible concentration of 2-piperidone (Fig. 4C). Hence, the gene *CILFYP54_00697* was identified as capable of catalyzing the dehydration and cyclization of 5AVA to produce 2-piperidone. We named this gene *avaC* (5-aminovaleric acid cyclase). Notably, co-expression of *avaC* and *ypdA* resulted in the production of 3.46 ± 0.02 mM 2-piperidone, significantly higher than expressing *avaC* alone (Fig. 4C), suggesting that the presence of *ypdA* might enhance the ability of *avaC* to convert 5AVA to 2-piperidone*.*

### Additional Genes in Gut Microbial Strains that Convert 5AVA to 2-Piperidone

**Fig. 5:**
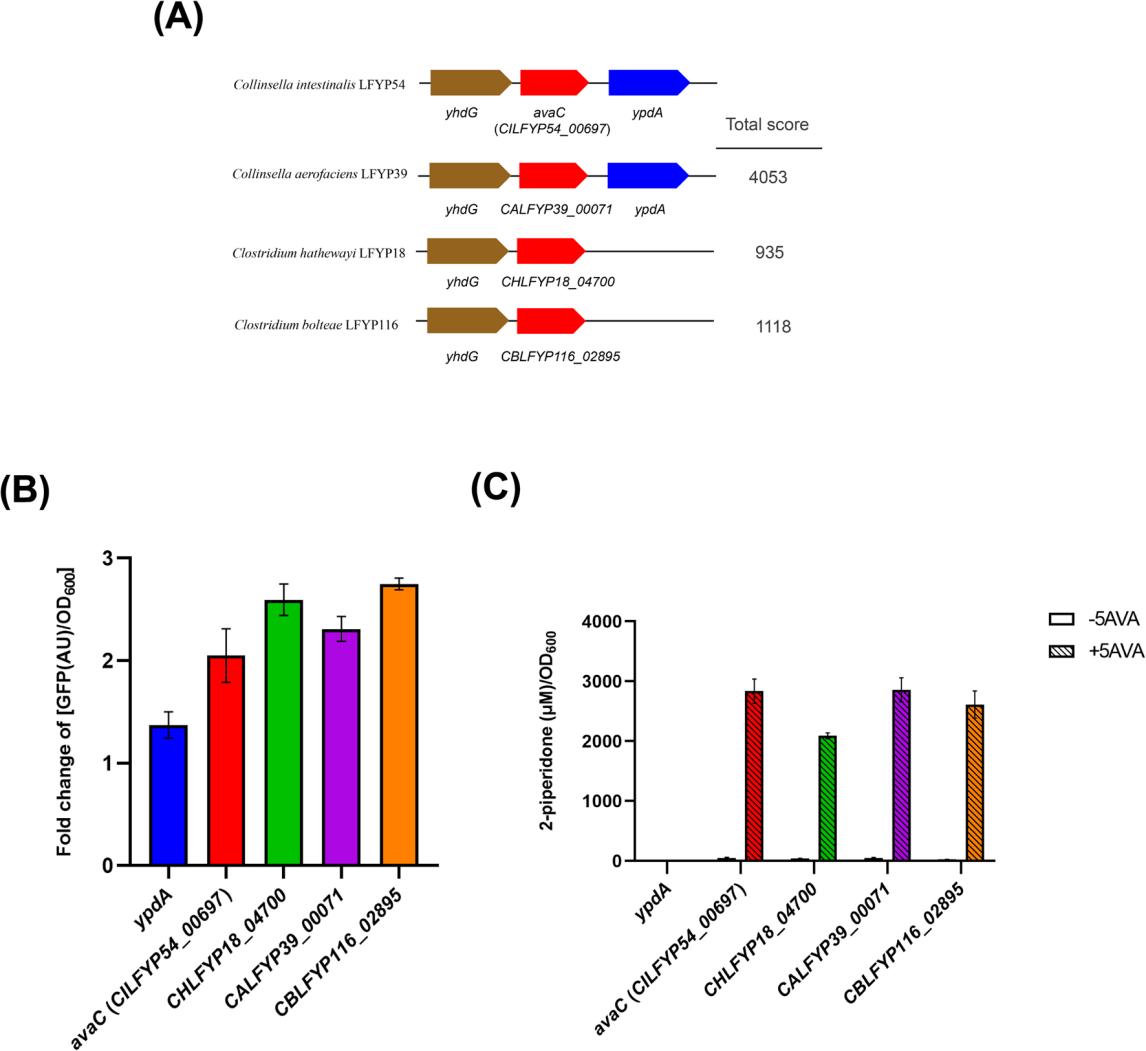
Homologous genes of *avaC* in other intestinal bacteria. (**A**) Similar gene clusters containing gene *avaC* from other intestinal bacteria. *yhdG* encodes putative amino acid permease. *avaC* (*CILFYP54_00697*) encodes 5-aminovaleric acid cyclase. Similarity score was calculated by Nucleotide Basic Local Alignment Search Tool (BLAST). (**B**) Fold change of [GFP(AU)/OD_600_] of *E. coli* strains with the two-plasmid biosensor system expressing the homologous genes as indicated. (**C**) Concentration of 2-piperidone per OD_600_ cell in the supernatant of *E. coli* strains expressing the homologous genes incubated without or with 5 mM 5AVA. Statistically significant differences between 2-piperidone concentrations in the strains carrying “*CILFYP54_00697*”, “*CHLFYP18_04700*”, “*CALFYP39_00071*”, and “*CBLFYP116_02895*”, respectively, and co-incubated with 5AVA were determined by One-way ANOVA and Tukey’s multiple comparison test. No significant differences were detected. For (B) and (C), error bars represent the standard error of mean from three biological replicates

We had identified *avaC*, which converted 5AVA to 2-piperidone in *C. intestinalis* LFYP54. Our next step was to characterize other genes with similar functionality as *avaC* in *C. aerofaciens* LFYP39, *C. bolteae* LFYP116, and *C. hathewayi* LFYP18. The nucleotide sequence of *avaC* from *C. intestinalis* LFYP54 was aligned with whole-genome sequences of the other three strains. *C. aerofaciens* LFYP39, phylogenetically related to *C. intestinalis* LFYP54, possessed a gene (*CALFYP39_00071*) sharing highly similar sequence with *avaC*. However, *C. hathewayi* LFYP18 and *C. bolteae* LFYP116, which were distantly related to *C. intestinalis* LFYP54, harbored genes (*CHLFYP18_04700* and *CBLFYP116_02895*, respectively) with more dissimilar sequences (Fig. 5A). Protein sequence alignment further confirmed the similarity of these three protein sequences to the *avaC* protein (Table S7). These proteins were annotated as a group of enzymes catalyzing the hydrolysis of a wide range of substrates bearing amide or ester functional groups at carbon (Pieper et al., 2009), representing the reverse of the cyclization reaction.

To assess the function of these additional genes, we cloned them to the production plasmid. Given that *ypdA* produced only a negligible concentration of 2-piperidone after incubation with 5AVA (Fig. 4C), it was utilized as a negative control in the subsequent experiment. The results demonstrated that the other three genes also possessed the function to convert 5AVA into 2-piperidone. The production levels of 2-piperidone did not exhibit significant differences across the four genes when incubated with 5AVA (Fig. 5B, C). Hence, we designated *CALFYP39_00071*, *CHLFYP18_04700,* and *CBLFYP116_02895* as *avaC* as well.

To further elucidate the distribution of this type of enzyme in bacteria, we conducted a sequence similarity search using the four *avaC* genes in the National Center for Biotechnology Information (NCBI) GenBank database (Table S6). The results showed that: (1) The homologous genes exhibited considerable overlap, with varying scores of sequence similarity among them; (2) These homologous genes were distributed in 5 phyla, primarily in Bacillota and Actinomycetota*;* (3) Bacteria containing homologous genes were widely distributed across various environments, with the majority belonging to gastrointestinal tract bacteria, while some were present in natural or engineered environments.

To assess whether strains in other environments possess the function to convert 5AVA to 2-piperidone, we randomly selected 5 predicted AvaC homologous proteins from strains distributed in various environments, including chicken gut, mud, termite gut, mud snails and soil (Table S8), and measured their ability to produce 2-piperidone in *E. coli* following the methods described above. The results demonstrated that the AvaC homologous protein from *Clostridium* sp. *KNHs214* isolated from soil could efficiently produce 2-piperidone and the homologous protein from *Atopobium* sp*. ICM42b* produced a low level of 2-piperidone (Fig. S4). The results suggested the existence of other high efficient 5-aminovaleric acid cyclases from the predicted AvaC homologous proteins in environmental bacterial strains.

## Discussion

In this study, we identified four human gut-derived bacterial strains, *C. intestinalis* LFYP54, *C. bolteae* LFYP116, *C. aerofaciens* LFYP39, and *C. hathewayi* LFYP18, which produced 2-piperidone from 5AVA. Additionally, we demonstrated that 2-piperidone could be produced from proline through cross-feeding between *C. difficile* LFYP43 and one of the four 2-piperidone producing strains respectively. Furthermore, a novel gene, *avaC*, was identified in the aforementioned four strains, showing the ability to convert 5AVA to 2-piperidone. Additionally, bioinformatic analysis unveiled the wide distribution of *avaC* among the natural environmental bacterial species.

2-piperidone is a precursor for the synthesis of nylon-5 (Han & Lee, 2023), and its biosynthesis approach has garnered high-profile attention. The development of efficient biocatalytic methods for producing 2-piperidone can advance environmentally friendly 2-piperidone production. To date, expressing different 5AVA cyclization enzymes, including Act, ORF26, and CaiC, in genetically modified strains under identical culture conditions resulted in similar 2-piperidone yields (Zhao et al., 2023). Compared to *orf*26, the newly discovered gene, *avaC*, exhibited an approximately 50-fold increase in yield in the cyclization of 5AVA to 2-piperidone in this study. Furthermore, in order to compare the efficiency between *avaC* and the previously reported 2-piperidone producing genes comprehensively in the heterologous expression system utilized in this study, we additionally expressed *act* and *caiC*. The results showed that the concentration of 2-piperidone produced by *avaC* is approximately 14-fold higher than that of *act* and the *caiC* gene was unable to produce detectable 2-piperidone levels in this system (Fig. S5). The cyclization step of 5AVA is rate-limiting to the yield of 2-piperidone (Zhao et al., 2023). Our results suggested that *avaC* may address the bottleneck of biological 2-piperidone synthesis. Furthermore, Act, ORF26, and CaiC can cyclize 4ABA and 6ACA to form four- and six-carbon lactams (Gordillo Sierra & Alper, 2020). These reactions involve intramolecular dehydration cyclization reactions of ω-amino acids. Four- and six-carbon lactams also hold significant industrial value. Whether *avaC* can cyclize other ω-amino acids requires further investigation.

In the *avaC* screening experiment mentioned above, although both ZQD5 and ZQD4 exhibited a higher fold change of [GFP (AU)/OD_600_] values compared to the other colonies (Fig. 3B), the production of 2-piperidone was obviously lower in ZQD5 after co-incubation with 5AVA (Fig. 3D). The primary reason might be that the two-plasmid system used in the experiment exhibited high sensitivity (Fig. S2). ZQD5 indeed produced minimal concentration of 2-piperidone after co-incubation with 5AVA, which might exceed the threshold required for inducing the GFP signal in pLacSens. The insertion sequence in ZQD5 might be a low-efficiency 5AVA cyclase under the current experimental conditions. The function of the insertion sequence in ZQD5 necessitates further identification in future studies.

**Fig. 6:**
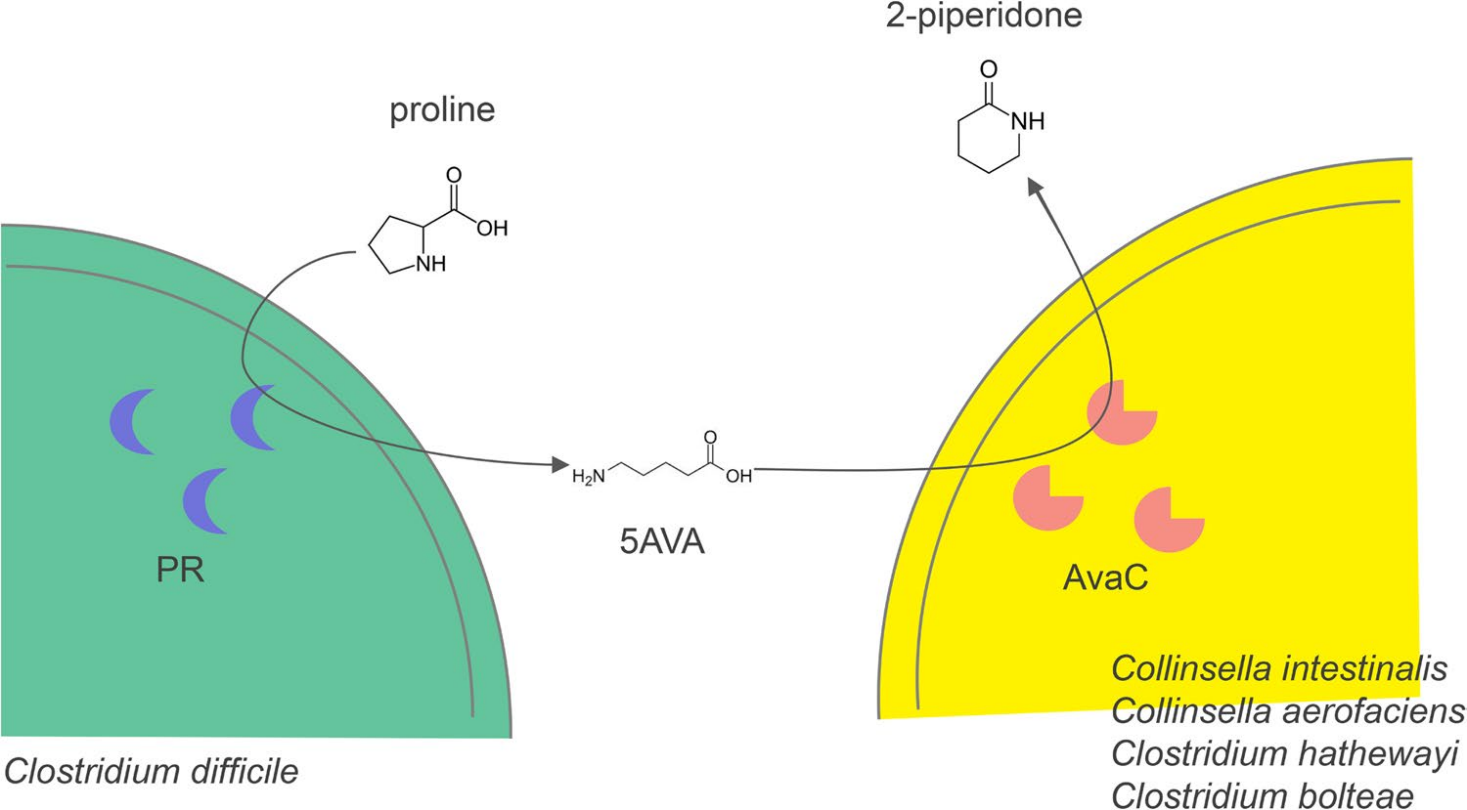
Schematic diagram of the cooperative metabolism of proline to 2-piperidone by gut bacterial strains. Proline is converted into 5AVA by the PR (proline reductase) enzyme in *C. difficile*. Subsequently, 5AVA is transformed into 2-piperidone by AvaC identified in *C. intestinalis*, *C. aerofaciens*, *C. hathewayi*, or *C. bolteae*

Interestingly, 2-piperidone has been identified as a biomarker for various diseases, including epithelial ovarian cancer (EOC), inflammatory bowel disease (IBD), esophageal squamous cell carcinoma (ESCC), and others (Ahmed et al., 2016; Chen et al., 2020; Ke et al., 2015; Xuan et al., 2020). However, the biological function of 2-piperidone, and its sources in vivo remain largely unclear. Our study unveils the ability of intestinal bacterial isolates to produce 2-piperidone in vitro. In particular, a cooperative metabolic pathway exists between *C. difficile* and the 2-piperidone producing strains: *C. difficile* metabolizes proline into 5AVA via the PR (proline reductase) enzyme (Bouillaut et al., 2013; Jackson et al., 2006) and subsequently *C. intestinalis*, *C. bolteae*, *C. aerofaciens*, or *C. hathewayi* utilizes AvaC to convert 5AVA produced by *C. difficile* into 2-piperidone (Fig. 6). Yet, whether 2-piperidone can be produced in vivo by gut microbiota and its potential impact on the host remain elusive. Future in vivo experimentation is required to elucidate the relationship between 2-piperidone, gut microbiota, and host physiology.

### Supplementary Information

Below is the link to the electronic supplementary material.

Supplementary file1 (PDF 811 KB)

Supplementary file2 (XLSX 118 KB)

## Data Availability

Data are available with reasonable requirements.
