# Supplementary material for: Identification of avaC from Human Gut Microbial Isolates that Converts 5AVA to 2-Piperidone
Source: J Microbiol. 2024 Jun 17;62(5):367–79. doi: 10.1007/s12275-024-00141-0 (PMC11196342; doi:10.1007/s12275-024-00141-0)
Supplement: Supplementary file 1 — Supplementary file1 (PDF 811 KB) [file 12275_2024_141_MOESM1_ESM.pdf]

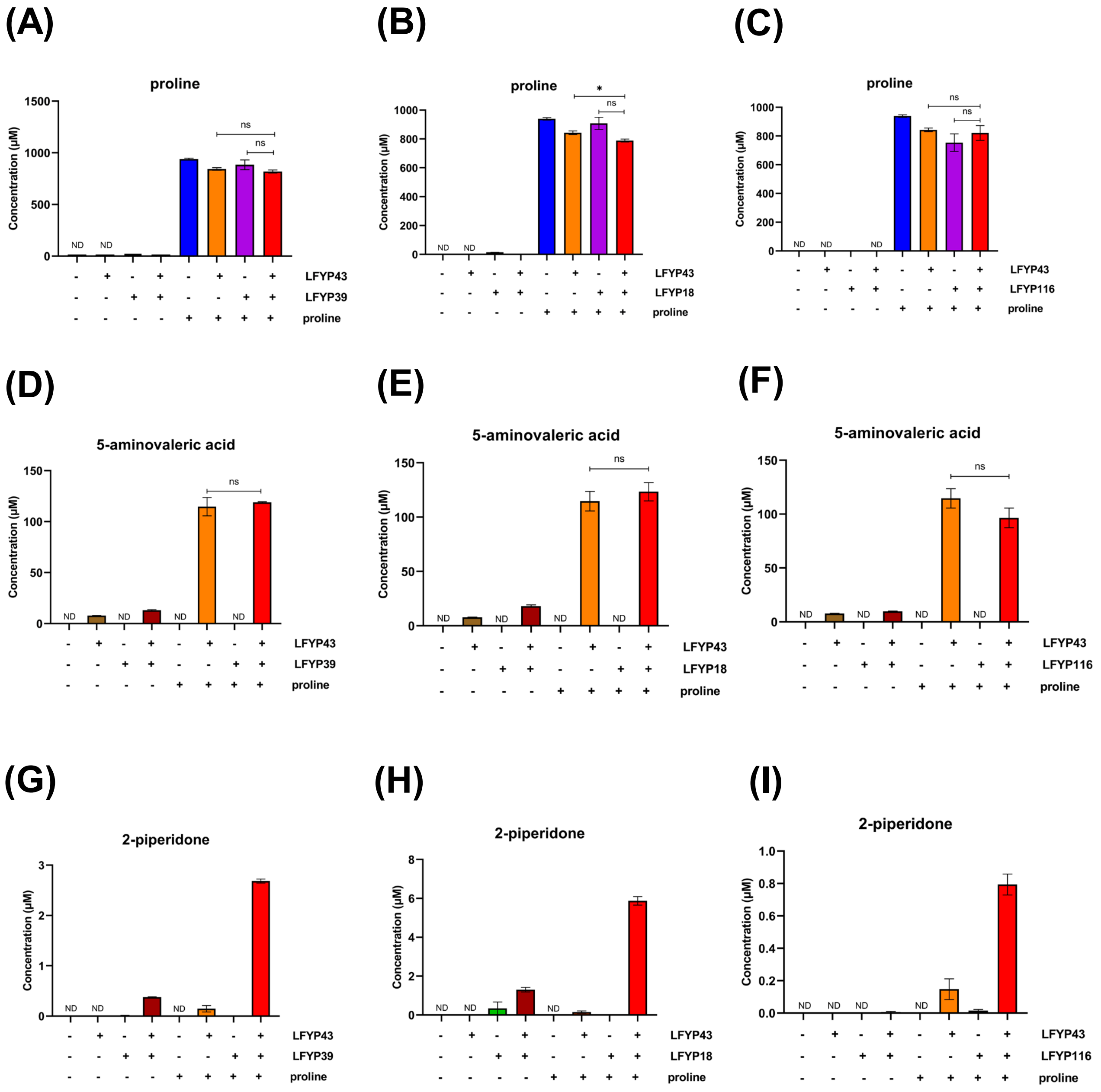

Fig. S1. Metabolism of proline into 2-piperidone by gut bacterial strains. Concentrations of proline (A–C), 5AVA (D–F), and 2-piperidone (G–I) in the supernatant of *C. difficile* LFYP43 and *C. aerofaciens* LFYP39, *C. hathewayi* LFYP18, or *C. bolteae* LFYP116 under different incubation conditions. In (A–F), statistical analysis was conducted to compare the concentrations of proline (A–C) and 5AVA (D–F) under specific conditions. For (A–F), the statistical significance was determined by Student's t-test (unpaired, two-tailed). ND, not detected; $*p < 0.05$. For (A–I), error bars represent the standard error of mean from three biological replicates.

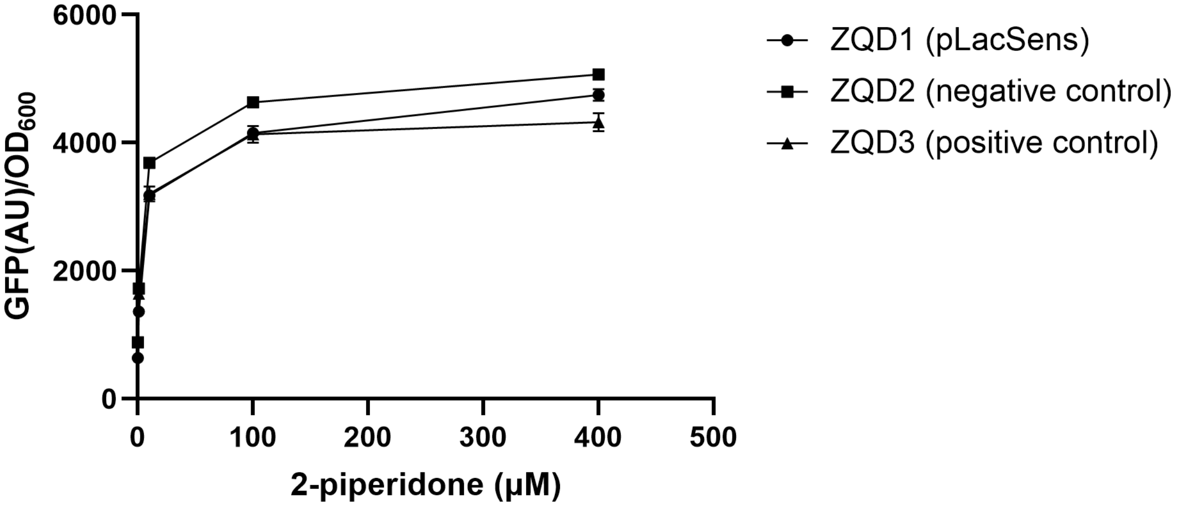

Fig S2. Sensitivity of the two-plasmid biosensor system to 2-piperidone. GFP(AU)/OD_{600} values of *E. coli* DH10B carrying different plasmids in response to various concentrations of 2-piperidone. ZQD1(pLacSens); ZQD2 (negative control), pLacSens and an empty production plasmid; ZQD3 (positive control), pLacSens and a production plasmid with *orf26*. Error bars represent the standard error of mean from three biological replicates.

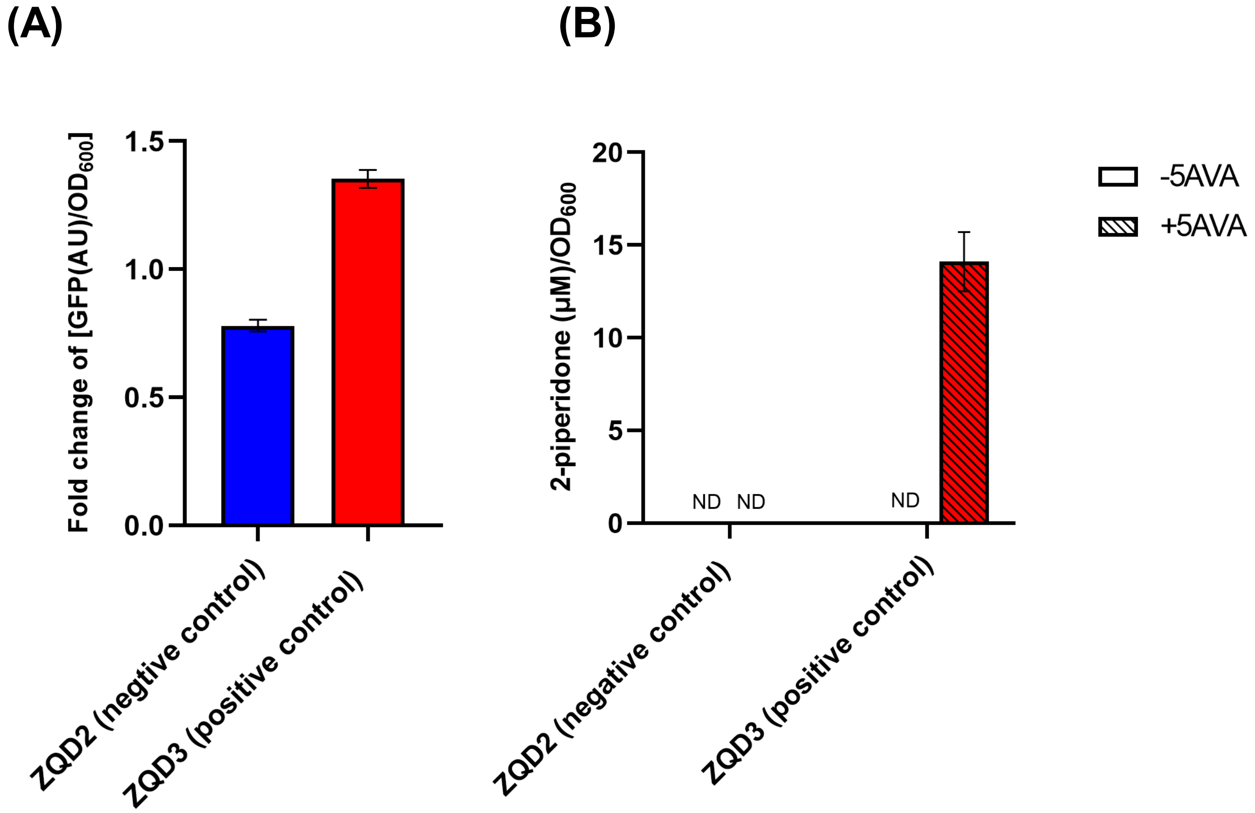

Fig S3. Control groups for the two-plasmid screening system. (A) Fold change of [GFP(AU)/OD_{600}] values of the positive control and negative control strains. (B) Concentration of 2-piperidone per OD_{600} cell in the supernatant of the positive control and negative control strains incubated without or with 5 mM 5AVA. For (A) and (B), error bars represent the standard error of mean from three biological replicates.

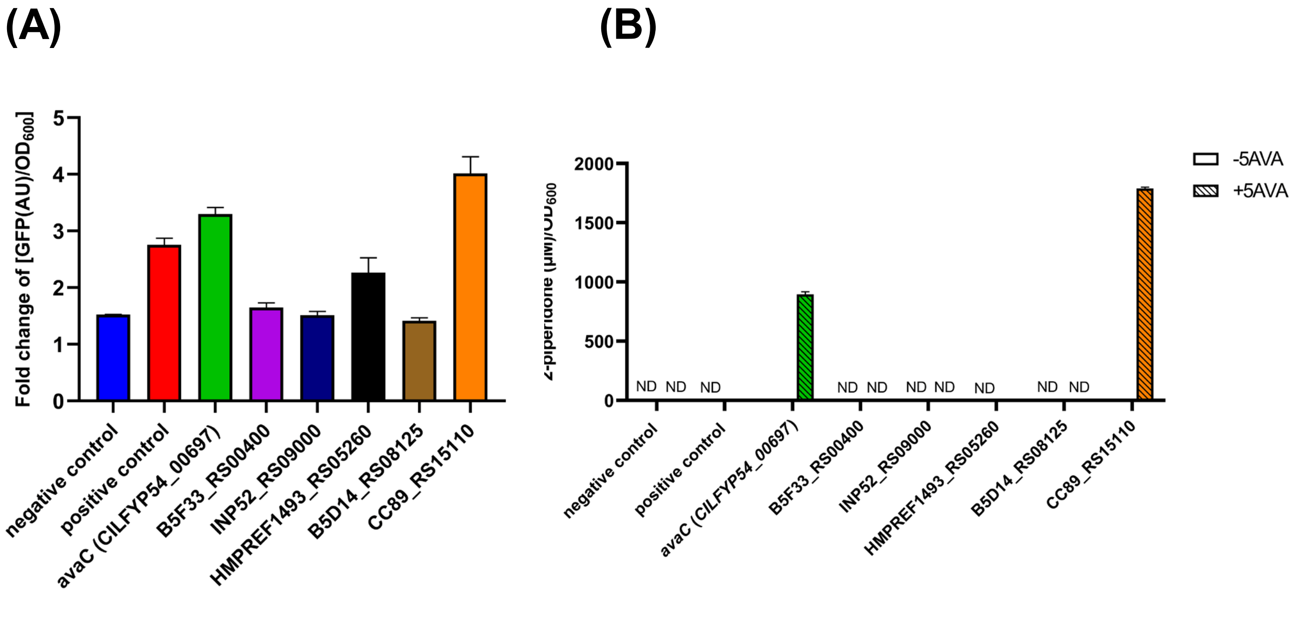

Fig S4. Functionality test of *avaC* homologous genes from various environmental strains. (A) Fold change of [GFP(AU)/OD_{600}] values of *E. coli* carrying the *avaC* homologous genes in various environmental strains. (B) Concentration of 2-piperidone per OD_{600} cell in the supernatant of *E. coli* carrying the *avaC* homologous genes in selected environmental strains incubated without or with 5 mM 5AVA. For (A) and (B), error bars represent the standard error of mean from three biological replicates; *B5F33_RS00400*, *INP52_RS09000*, *HMPREF1493_RS05260*, *B5D14_RS08125* and *CC89_RS15110* represents *avaC* homologous genes from *Collinsella* sp. *An2* (chicken gut), *Thermophilibacter immobilis* (mud), *Atopobium* sp. *ICM42b* (termite gut), *Anaerorhabdus furcosa* (mud snails), and *Clostridium* sp. *KNHs214* (soil) respectively.

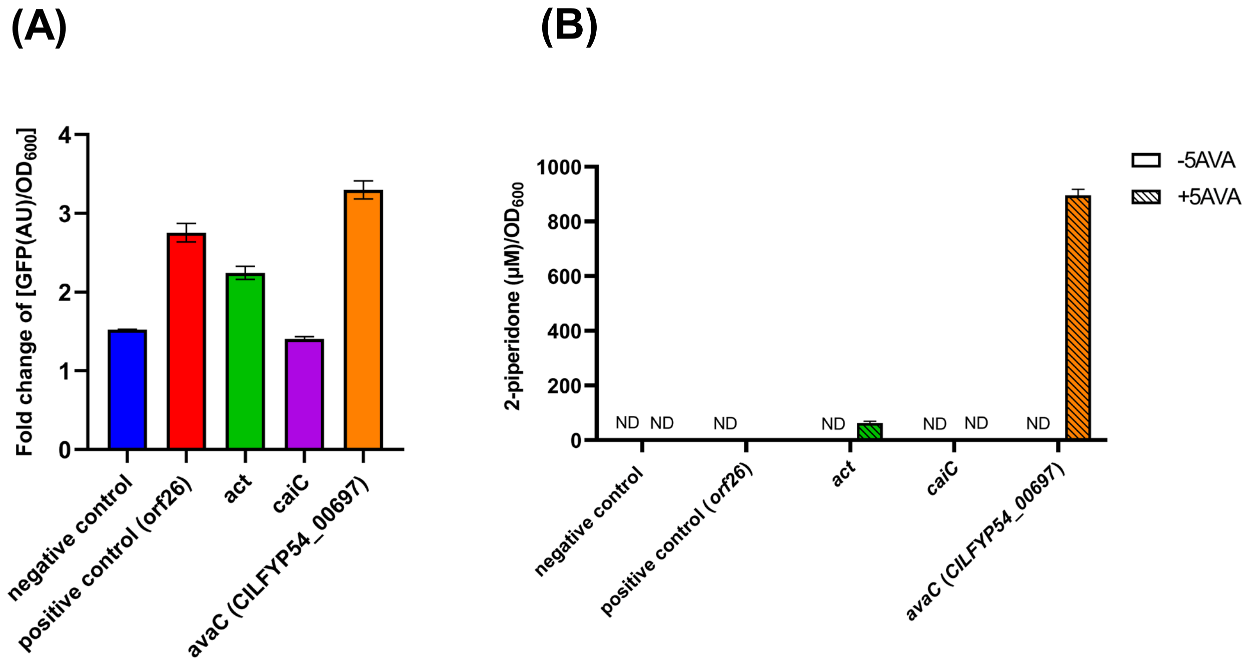

Fig S5. Comparison of the efficiency of *avaC* to previously reported 2-piperidone producing genes. (A) Fold change of [GFP(AU)/OD_{600}] values of *E. coli* expressing various 2-piperidone producing genes. (B) Concentration of 2-piperidone per OD_{600} cell in the supernatant of *E. coli* carrying various 2-piperidone producing genes incubated without or with 5 mM 5AVA. For (A) and (B), error bars represent the standard error of mean from three biological replicates; *act*, β-alanine CoA transferase; *caiC*, crotonobetaine CoA ligase.
